# CAPS1 Negatively Regulates Hepatocellular Carcinoma Development through Alteration of Exocytosis-Associated Tumor Microenvironment

**DOI:** 10.3390/ijms17101626

**Published:** 2016-09-27

**Authors:** Ruyi Xue, Wenqing Tang, Pingping Dong, Shuqiang Weng, Lijie Ma, She Chen, Taotao Liu, Xizhong Shen, Xiaowu Huang, Si Zhang, Ling Dong

**Affiliations:** 1 Department of Gastroenterology and Hepatology, Shanghai Institute of Liver Diseases, Zhongshan Hospital of Fudan University, Shanghai 200032, China; wanjin1986@163.com (W.T.); pingdongqy@163.com (P.D.); weng.shuqiang@zs-hospital.sh.cn (S.W.); liu.taotao@zs-hospital.sh.cn (T.L.); shen.xizhong@zs-hospital.sh.cn (X.S.); 2 Department of Hepatic Surgery of Liver Cancer Institute, Zhongshan Hospital, Fudan University, Shanghai 200032, China; 14211210033@fudan.edu.cn (L.M.); huang.xiaowu@zs-hospital.sh.cn (X.H.); 3 Key Laboratory of Glycoconjugate Research Ministry of Public Health, Department of Biochemistry and Molecular Biology, Shanghai Medical College, Fudan University, Shanghai 200032, China; shechen@fudan.edu.cn (S.C.); zhangsi@fudan.edu.cn (S.Z.)

**Keywords:** calcium-dependent activator protein for secretion 1, exocytosis, hepatocellular carcinoma, membrane trafficking, tumor microenvironment, prognosis

## Abstract

The calcium-dependent activator protein for secretion 1 (CAPS1) regulates exocytosis of dense-core vesicles (DCVs) in neurons and neuroendocrine cells. The role of CAPS1 in cancer biology remains unknown. The purpose of this study was to investigate the role of CAPS1 in hepatocellular carcinoma (HCC). We determined the levels of CAPS1 in eight hepatoma cell lines and 141 HCC specimens. We evaluated the prognostic value of CAPS1 expression and its association with clinical parameters. We investigated the biological consequences of CAPS1 overexpression in two hepatoma cell lines in vitro and in vivo. The results showed that loss of CAPS1 expression in HCC tissues was markedly correlated with aggressive tumor phenotypes, such as high-grade tumor node metastasis (TNM) stage (*p* = 0.003) and absence of tumor encapsulation (*p* = 0.016), and was associated with poor overall survival (*p* = 0.008) and high recurrence (*p* = 0.015). CAPS1 overexpression inhibited cell proliferation and migration by changing the exocytosis-associated tumor microenvironment in hepatoma cells in vitro. The in vivo study showed that CAPS1 overexpression inhibited xenograft tumor growth. Together, these results identified a previously unrecognized tumor suppressor role for CAPS1 in HCC development.

## 1. Introduction

Liver cancer is the fifth most frequently diagnosed cancer, and the second most frequent cause of cancer death worldwide [1]. Of primary liver cancers, hepatocellular carcinoma (HCC) represents the most common histological subtype, accounting for 70%–85% of the total liver cancer occurrence worldwide [2]. HCC is asymptomatic in its early stages, and even after tumor resection the survival and recurrence are extremely discouraging. Prognostic prediction is a vital component in clinical management of HCC patients. However, current clinicopathologic factors, such as α-fetoprotein (AFP), tumor node metastasis (TNM) stage, and Barcelona clinic liver cancer (BCLC) stage, cannot accurately predict the outcome of HCC patients. It is important to identify novel prognostic factors for HCC [3].

Calcium-dependent secretion activator 1 (CAPS1) was identified in 1992 as a novel cytosolic brain protein required for Ca^2+^-activated secretion in two permeable neuroendocrine cell types [4]. The protein is a dimer of 145 kDa subunits, exhibits Ca^2+^-dependent interaction with hydrophobic matrix, and binds to phospholipid vesicles, suggesting a membrane-associated function. CAPS1 exists in a variety of species; in vertebrates, CAPS1 has a closely related isoform called CAPS2 [5,6,7]. The CAPS family regulates secretory granule exocytosis, including monoamines and neuropeptides, as well as Golgi trafficking. It is well recognized that CAPS1 encodes a 1353-amino acid protein with four functional domains including a C2 domain that binds calcium, a pleckstrin homology (PH) domain that binds to phosphatidylinositol-(4,5)-bisphosphate (PIP2), a Munc homology domain (MHD) involved in secretion, and a dense core vesicle-binding domain from N-terminal to C-terminal [7].

CAPS1 was independently discovered in *Caenorhabditis elegans* as the *unc-31* gene which is required for synaptic transmission [8] and in PC12 cells as an essential component of the Ca^2+^-triggering machinery for exocytosis [9]. Though CAPS1 is primarily expressed in neurons and neuroendocrine cells in the murine species, nonneuronal tissues such as the liver contain a certain level of CAPS1 [4,7].

Up to now, only a few studies have been conducted on the potential connection between CAPS1 and human cancers. Loss of CAPS1, identified in primitive neuroectodermal tumors of the central nervous system by single nucleotide polymorphism (SNP) and quantitative PCR, was reported to be associated with a poor prognosis [10]. We previously identified CAPS1 as a promising serum biomarker for HCC diagnosis by magnetic bead-based (MB) and matrix-assisted laser desorption/ionization time-of-flight mass spectrometry (MALDI-TOF-MS) [11]. In the present work, we further studied the prognostic value and biological function of CAPS1 in hepatocellular carcinoma.

## 2. Results

### 2.1. Reduced CAPS1 Expression in HCC Tissues and HCC Cells

**Figure 1:**
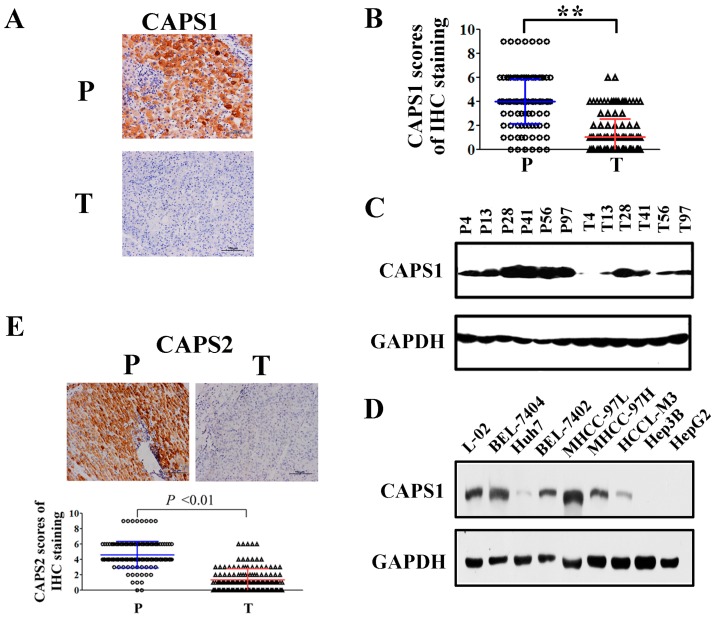
Loss of calcium-dependent activator protein for secretion 1 (CAPS1) expression in clinical hepatocellular carcinoma (HCC) tissues and HCC cell lines. (**A**) Typical patterns of CAPS1 staining in paired HCC tissue specimens. P, peritumoral tissues; T, tumor tissues; (**B**) Scores of immunochemistry staining of CAPS1 in 141 HCC patients. The mean composite score of immunohistochemical (IHC) staining of peritumoral tissues is four (in blue color), while that of tumor tissues is one (in red color). ** *p* < 0.01; (**C**) Protein expression of CAPS1 in tumor tissues with paired peritumor tissues of six random HCC cases; (**D**) Protein levels of CAPS1 in a normal liver cell line (L-02) and cultured HCC cell lines. The experiment was repeated three times and representative samples are shown; (**E**) Typical patterns of CAPS2 staining in paired HCC tissue specimens (**upper** panel) and scores of immunochemistry staining of CAPS2 in 141 HCC patients (**bottom** panel). The mean composite score of IHC staining of peritumoral tissues is 4.6 (in blue color), while that of tumor tissues is 1.3 (in red color).

We performed immunohistochemistry analysis to determine the expression and the location of CAPS1 in tumor tissues and paired non-tumor liver tissues from 141 HCC patients who had undergone curative resection. CAPS1-positive signals mainly localized in the cytoplasm (Figure S1). CAPS1 protein expression was significantly decreased in the tumor tissues compared with peritumoral tissues (Figure 1A), as demonstrated by paired *t* test (*p* < 0.001) (Figure 1B). Loss of CAPS1 compared with peritumoral tissues was perceived in 85% (120 of 141) of HCC samples, compared with peritumoral tissues. Also, six paired HCC samples were selected randomly and detected by Western blotting, validating this finding (Figure 1C). CAPS1 expression was also determined by Western blotting in eight HCC cell lines, as well as a normal liver cell line, L-02 (Figure 1D). Low CAPS1 expression was observed in most HCC cell lines except BEL-7404 and MHCC-97L cells. Moreover, in HCC cell lines with stepwise increased metastatic potential (MHCC-97L < MHCC-97H < HCCL-M3) [12], the expression of CAPS1 decreased gradually (MHCC-97L > MHCC-97H > HCCL-M3). We analyzed the genomic alterations of CAPS1 gene in 260 HCC cases from the International Cancer Genome Consortium (ICGC) [13]. CAPS1 showed somatic mutations in 203 of the 260 HCC cases (78.08%), indicating that CAPS1 somatic mutation may contribute to CAPS1 downregulation in HCC. Similar to CAPS1, CAPS2 protein expression was significantly decreased in the tumor tissues compared with peritumoral tissues (Figure 1E).

### 2.2. Decreased CAPS1 Expression Was Associated with Aggressive Tumor Phenotypes in HCC Patients

We next investigated the association between CAPS1 expression and patients’ clinicopathologic characteristics in this cohort of 141 patients. As summarized in Table 1, decreased CAPS1 expression was associated with aggressive tumor phenotypes, such as high-grade TNM stage (*p* = 0.003) and absence of tumor encapsulation (*p* = 0.016), as indicated by the chi-square test. However, the expression failed to be associated with other clinical pathological factors such as gender, age, hepatitis B surface antigen, hepatitis C virus (HCV), preoperative AFP, liver cirrhosis, BCLC stage, tumor size, tumor number, vascular invasion, and tumor differentiation.

### 2.3. Decreased CAPS1 Expression Correlated with Poor Prognosis of HCC

We further investigated the prognostic value of CAPS1 in this cohort of 141 patients. Univariate analysis suggested that decreased CAPS1 expression in tumor tissue was significantly associated with decreased overall survival (OS, *p* = 0.014) and shorter time to recurrence (TTR, *p* = 0.019) in HCC patients (Table 2). When the variables that were found to be meaningful in univariate analysis were combined in multivariate analysis, CAPS1 expression in tumor tissue remained as independent prognostic factors in HCC patients for both OS (*p* = 0.010, hazard ratio (HR), 0.209) and TTR (*p* = 0.007, HR, 0.324) (Table 3).

**Figure 2:**
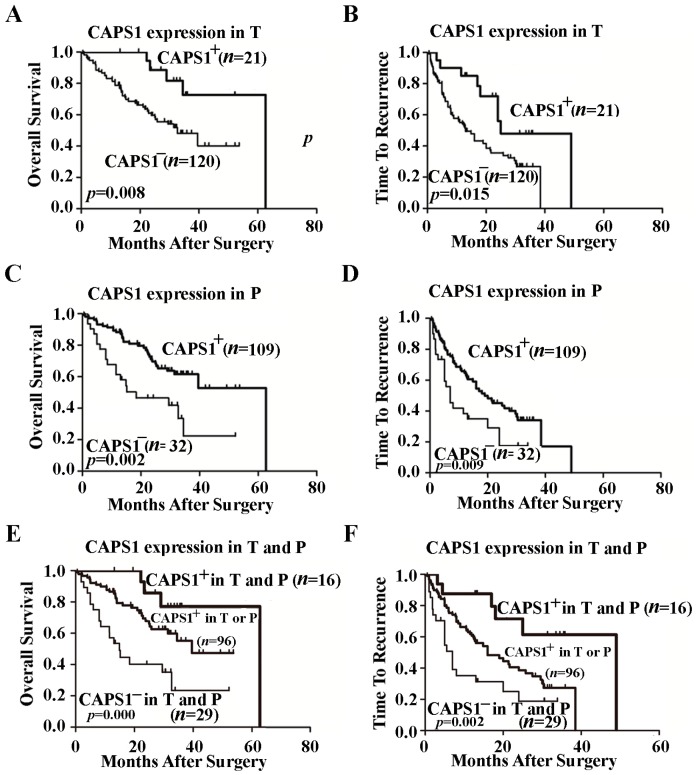
Kaplan–Meier curves of the overall survival (OS) and time to recurrence (TTR) of 141 HCC patients who underwent curative surgery (*p* values were obtained by log-rank test). (**A**,**B**) Kaplan–Meier curves for OS (**A**) and TTR (**B**) according to the scores of CAPS1 in tumor tissues (T); (**C**,**D**) Kaplan–Meier curves for OS (**C**) and TTR (**D**) according to the scores of CAPS1 in peritumoral tissue (P); (**E**,**F**) Kaplan–Meier curves for OS (**E**) and TTR (**F**) according to the combined scores of CAPS1 in T and P.

Kaplan–Meier analysis and the log-rank test showed that patients with negative CAPS1 expression in tumor tissues had a significantly shorter median OS and TTR (OS, 30.8 months; TTR, 18.3 months) than those with positive CAPS1 expression (OS, 53.4 months, *p* = 0.008; TTR, 32.6 months, *p* = 0.015) (Figure 2A,B). Similarly, negative CAPS1 expression in peritumoral tissues was also significantly associated with shorter OS (*p* = 0.002) and TTR (*p* = 0.009) (Figure 2C,D). In addition, based on the positive and negative CAPS1 expression in tumor tissues and peritumoral tissues, all 141 cases were classified into three groups: both positive, positive CAPS1 expression in both tumor tissues and peritumoral tissues; one of the two positive, positive CAPS1 expression in either tumor tissues or peritumoral tissues; both negative, negative CAPS1 expression in both tumor tissues and peritumoral tissues. Patients with both positive CAPS1 expression had the best prognosis in the three groups, while those with both negative CAPS1 expression had the worst (OS, *p* = 0.000; TTR, *p* = 0.002) (Figure 2E,F).

### 2.4. Overexpression of CAPS1 Inhibited Cell Growth and Migration, Decreased the Number of Membrane-Enclosed Vesicles and Altered Tumor Microenvironment in HCC Cell Lines

**Figure 3:**
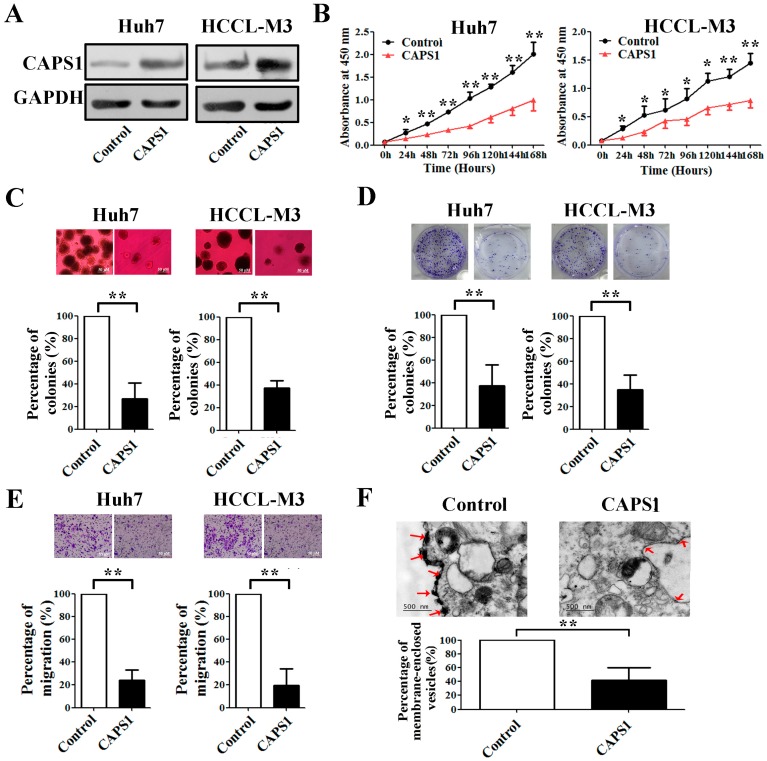
CAPS1 overexpression reduced cell proliferation and migration and inhibited exocytosis in HCC cells. (**A**) CAPS1 overexpression efficiency was verified by Western blotting in Huh7 and HCCLM3 cells; (**B**) Cell growth curves of Huh7 and HCCLM3 cells transfected with control vector or CAPS1; (**C**–**E**) Soft agar colony assay (**C**), Plate colony formation assay (**D**) and transwell migration assay (**E**) for Huh7 and HCCLM3 cells transfected with control vector or CAPS1; (**F**) Representative electron microscopy images of Huh7 cells transfected with control vector or CAPS1. Arrow points: membrane-enclosed vesicles. Representative pictures are shown in top panels. Quantitative results of at least three independent replicates are shown in bottom panels. * *p* < 0.05; ** *p* < 0.01.

We constructed stable CAPS1-overexpressing clones by transfecting Huh7 and HCCLM3 cells with pCMV6 Entry/myc-CAPS1 plasmid and screening with G418. The overexpression efficiency was confirmed by Western blotting (Figure 3A). The cell growth was determined by cell counting kit-8 (CCK-8) and colony formation assays. Overexpression of CAPS1 significantly decreased cell viability (Figure 3B) and inhibited colony formation (Figure 3C,D) in both Huh7 and HCCLM3 cells, which indicated that CAPS1 inhibited the in vitro proliferation of HCC cells. In addition, transwell assays demonstrated that overexpression of CAPS1 substantially repressed the migratory ability of Huh7 and HCCLM3 cells (Figure 3E). Moreover, ultrastructural analysis revealed that overexpression of CAPS1 decreased the number of membrane-enclosed vesicles in Huh7 cells (Figure 3F), which indicated that CAPS1 inhibited the exocytosis process of HCC cells.

**Figure 4:**
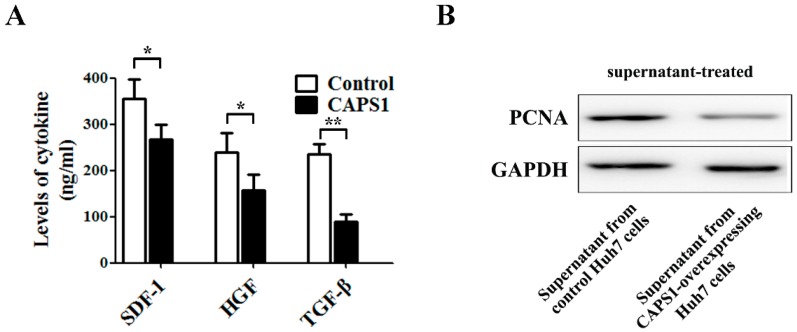
CAPS1 overexpression changed microenvironment in HCC cells. (**A**) The levels of stromal cell-derived factor 1 (SDF-1), hepatocyte growth factor (HGF), and transforming growth factor-beta (TGF-β) were determined by enzyme-linked immunosorbent assay (ELISA) in culture supernatant derived from Huh7 cells stably transfected with control vector or CAPS1; (**B**) Huh7 cells were treated with the culture supernatant from Huh7 cells stably transfected with control vector or CAPS1. Forty-eight hours later, the expression of proliferating cell nuclear antigen (PCNA), a marker of cellular proliferation, was detected by Western blotting. GAPDH: glyceraldehyde 3-phosphate dehydrogenase. * *p* < 0.05; ** *p* < 0.01.

It is well-known that the exocytosis process is closely related to the tumor microenvironment. To test whether CAPS1 could change the tumor microenvironment, we measured the concentration of stromal cell-derived factor 1 (SDF-1), hepatocyte growth factor (HGF), and transforming growth factor-beta (TGF-β) in the culture supernatant of Huh7 cells. The enzyme-linked immunosorbent assay (ELISA) showed that overexpression of CAPS1 decreased the levels of SDF-1, HGF, and TGF-β in the culture supernatant (Figure 4A). We further incubated untransfected Huh7 cells with the culture supernatant from Huh7 cells stably transfected with control vector or CAPS1. We detected the level of proliferating cell nuclear antigen (PCNA), a proliferation marker, by Western blotting. The level of PCNA was strongly decreased by the treatment with culture supernatant from Huh7 cells overexpressing CAPS1 (Figure 4B).

### 2.5. Overexpression of CAPS1 Reduces Tumor Growth in Xenograft-Bearing Nude Mice

**Figure 5:**
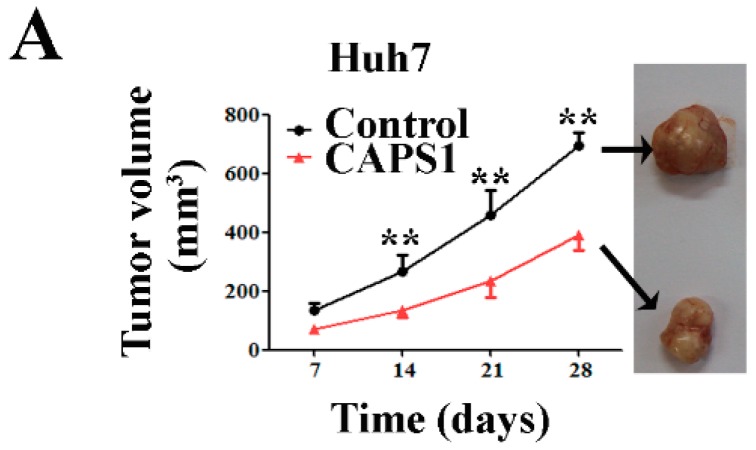

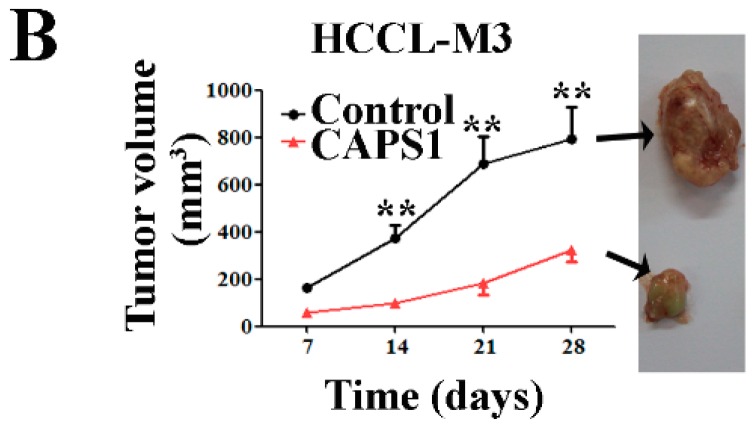
CAPS1 expression decreased tumor formation of hepatoma cells in vivo in nude mice. In vivo subcutaneous tumor growth curves and representative images of harvested subcutaneous tumors of Huh7 cells (**A**) and HCCLM3 cells (**B**) stably transfected with control vector or CAPS1 (*n* = 6). ** *p* < 0.01 vs. CAPS1 group.

We conducted a xenograft model by injecting the control and stable CAPS1-overexpressing clones subcutaneously into nude mice. CAPS1 overexpression significantly inhibited tumor growth for both Huh7 and HCCLM3 tumor cells, as assessed by tumor volume (Figure 5A,B). These results indicate that overexpression of CAPS1 expression in Huh7 and HCCLM3 cells markedly suppressed their tumorigenicity in vivo in mice.

## 3. Discussion

Miller et al. [10] identified *CAPS1* as a novel gene potentially involved in the tumorigenesis of neuroectodermal tumors by high-resolution genome-wide analysis of a large pineoblastoma cohort. Previously, we found that CAPS1 could be used as a serum biomarker for HCC diagnosis by the MB-based MALDI-TOF-MS approach [11], which implied that CAPS1 may have also participated in the tumorigenesis of endodermal tumors. In the present study, we added the new findings on the role of CAPS1 in HCC. Our results showed a significant loss of CAPS1 expression in HCC tissues and HCC cells, and a close correlation of reduced CAPS1 expression in the tumor tissues with poor survival and high recurrence rates in HCC patients. Reduced CAPS1 expression represented an independent prognostic factor. CAPS1 overexpression significantly inhibited tumor biological behavior, including cell proliferation and migration in vitro. This effect could be mediated by suppression of the exocytosis process and alteration of the tumor microenvironment in hepatoma cells. The in vivo study also showed that CAPS1 overexpression inhibited xenograft tumor growth in nude mice.

CAPS1 has been reported to be closely related to exocytosis of vesicles which contain neurotransmitters or peptides/neuromodulators in neurons, neuroendocrine cells, and endocrine cells [14,15]. To some extent, the liver also functions as an important secretory and endocrine organ. Hepatoma cells can secret cytokines, growth factors, and hormones such as TGF-β, SDF-1, and HGF, which are engaged in the HCC microenvironment [16]. It is now clear that these cytokines function in many aspects of tumor biology, including proliferation, angiogenesis, and invasion/metastasis [17]. High expression of hepatic vascular endothelial growth factor and hepatic platelet-derived endothelial cell growth factor was found to relate to poor prognosis in AFP-negative hepatocellular carcinoma patients after curative resection [18]. Hepatoma cell-derived insulin-like growth factor and fibroblast growth factor have the ability to act as autocrine growth factors for hepatoma cells and as potent paracrine mediators of angiogenesis to promote tumorigenesis and metastasis [19]. Secretory granule exocytosis is responsible for the secretion of peptides and proteins in hepatoma cells. Docking of secretory vesicles at the plasma membrane is a prerequisite step for exocytosis [20]. In the present study, we found that CAPS1 inhibited docking of secretory vesicles at the plasma membrane and decreased protumorigenic cytokines in hepatoma cells. We hence propose that CAPS1 interferes with the exocytosis of hepatic vesicles, and consequently, changes the secretion of certain cytokines, hormones, or serum proteins. As a result, an altered tumor microenvironment may contribute to malignant transformation. However, the present work is only a preliminary study which provides a starting point to investigate the link between CAPS1 and the tumor microenvironment. The mechanism by which CAPS1 acts as a tumor suppressor and the relationship between CAPS1-mediated microenvironmental changes and tumor cell biology still needs further investigation.

It is found that CAPS1 increases vesicle exocytosis by promoting trans-SNARE complex formation in neurons and neuroendocrine cells [21]. However, in our study, unlike the CAPS1 signaling in neurons and neuroendocrine cells, we found that CAPS1 decreased the number of membrane-enclosed vesicles in hepatoma cells. We also found that CAPS1 decreased the levels of SDF-1, HGF, and TGF-β in hepatoma cells. Therefore, CAPS1-mediated signal transduction may vary in different types of cells and also in different regulatory mechanisms. Previous studies have reported that CAPS is a cytosolic protein essential for the Ca^2+^-dependent fusion of dense-core vesicles (DCVs) in the plasma membrane [22]. We cannot exclude the possibility that the novel CAPS1 function reported in our manuscript is also Ca^2+^-dependent.

In summary, our data show that loss of CAPS1 expression is closely correlated with aggressive tumor phenotypes and poor prognosis in HCC patients. Our study also highlights a possibility that CAPS1 suppresses cell proliferation and migration via modulation of the exocytosis-associated tumor microenvironment in hematoma cells.

## 4. Materials and Methods

### 4.1. Patients

Ethical approval for human subjects was obtained from the Research Ethics Committee of Zhongshan Hospital (date of approval, 13 October 2014; permission code, 2014082), and informed consent was obtained from each patient. We retrospectively collected 141 patients undergoing liver cancer surgical resection in Zhongshan Hospital between October 2006 and February 2008. Collection of clinical data and postoperative follow-up were consistent with the harmonized standard [23]. Clinicopathologic characteristics are listed in the Table S1. All the subjects were followed up every three months for survival and recurrence inquiry, until death, contact failure, or until the end of the investigation, i.e., 31 March 2011, with the median period of OS 21 months (1–62.7 months) and TTR 14 months (1–49 months). OS was taken as the period ranging from the primary surgery to death or the last follow-up, and TTR was defined as the interval from the primary surgery to the first recurrence confirmed by AFP test, ultrasound, or computed tomography (CT).

### 4.2. Western Blotting

Protein expression of CAPS1 and PCNA was detected by Western blotting, as previously described [24]. Briefly, cells were lysed using radioimmunoprecipitation assay (RIPA) buffer and subjected to immunoblotting using anti-CAPS1 (PRS4561; Sigma-Aldrich, St. Louis, MO, USA), anti-PCNA (AB92552; Abcam, Cambridge, UK), and anti-GAPDH (AB181603; Abcam) antibodies. Proteins were visualized using an enhanced chemiluminescence kit (Pierce, Brookline, MA, USA).

### 4.3. Immunohistochemistry and Evaluation

HCC slides were incubated overnight at 4 °C with goat anti-human CAPS1 antibody (sc-135028; Santa Cruz Biotechnology, Santa Cruz, CA, USA) or goat anti-human CAPS2 antibody (M-15; Santa Cruz Biotechnology). Afterwards the specimens were incubated with a peroxidase-labeled polymer conjugated to donkey anti-goat IgG (Gene Tech Company Limited, Hong Kong, China) for 30 min. They were washed again, and 3,3′-diaminobenzidine (DAB) was used as a chromogen to visualize the reaction. The reaction was then kept inactive in H_2_O for 5 min, and the HCC slides were counterstained with hematoxylin, dehydrated, and sealed with cover slips. Negative controls were obtained by substituting primary antibodies with non-immune serum. The semiquantitative scoring system was based on both the staining intensity (0, negative; 1, weak; 2, intermediate; 3, strong) and the percentage of positive cells (0, 0% positive cells; 1, ≤25% positive cells; 2, 26%–50% positive cells; 3, >50% positive cells). The final score of each sample was obtained by multiplying the scores of staining intensity and percentage of positive cells [25]. We chose five randomized microscopic views of 400-fold magnification of each slide to score and get a composite score. Samples were classified as negative when the final scores were 0–3, and positive when 4–9. The evaluation of immunohistochemical staining was carried out by two independent pathologists who were unaware of the patient outcomes.

### 4.4. Establishment of Stable CAPS1 Overexpression Clones

L-02, BEL-7404, BEL-7402, Hep3B, HepG2, and Huh7 cell lines were purchased from the Type Culture Collection of the Chinese Academy of Sciences (Shanghai, China). MHCC-97L, MHCC-97H, and HCCL-M3 cell lines were purchased from the Liver Cancer Institute of Zhongshan Hospital, Fudan University (Shanghai, China). Expression vectors of CAPS1 or control vectors (Origene Technologies, Rockville, MD, USA) were transfected into Huh7 and HCCLM3 cells using lipofectamine 2000 reagent (Invitrogen, Carlsbad, CA, USA). Transfected Huh7 and HCCLM3 cells were screened by G418 (Sigma-Aldrich). The overexpression efficiency was confirmed by Western blotting.

### 4.5. Cell Proliferation, Growth and Migration Assay

Cell proliferation was measured using the Cell Counting Kit-8 (Dojindo Co., Kumamoto, Japan) according to the manufacturer’s instructions. Briefly, cells were incubated with CCK-8 for 1 h. Cell proliferation rate was assessed by measuring the absorbance at 450 nm with the Universal Microplate Reader (BIO-TEK Instruments, Minneapolis, MN, USA). Anchorage-independent growth ability was measured using the colony forming assay and soft agar colony formation assay, as previously [25]. The colonies with diameter >0.1 mm were counted and colony forming efficiency was calculated (Percentage of colonies = Number of colonies formed/Number of cells inoculated × 100%). Transwell migration assay was performed using 24-well format transwell chambers (8 mm pore filter, Corning, Canton, NY, USA), as previously [26]. Briefly, cells were added to the upper chamber of 24-well transwell chambers, and the lower chamber was filled with 600 μL of serum-free culture medium containing 20 μg/mL fibronectin. After incubation for 12 h, cells on the bottom surface of the membrane were fixed and stained with 0.1% crystal violet. Cells that had passed through the filter to the lower chamber were counted microscopically in 6 random regions per filter.

### 4.6. ELISA for Cytokine

Cultured media were collected from Huh7 cells cultured in serum-free Dulbecco’s modified Eagle’s medium (DMEM) and centrifuged at 1000× *g* for 5 min to obtain the supernatant. The level of SDF-1, HGF, and TGF-β in cultured media were determined by using human ELISA kits (R & D Systems, Minneapolis, MN, USA) according to the manufacturer’s instructions.

### 4.7. Transmission Electron Microscopy

Electron microscopy was conducted, as previously [27]. Briefly, cells were fixed with 2.5% glutaraldehyde and stored at 4 °C until embedding. Samples were postfixed with 1% osmium tetroxide, followed by dehydration with an increased concentration gradient of ethanol and propylene oxide. Samples were then embedded and ultrathin (50–60 nm) sections were cut using an ultramicrotome. Images were examined using a JEM-1200 electron microscope (JEOL, Tokyo, Japan) at 80 kV after the samples were stained with 3% uranyl acetate and lead citrate.

### 4.8. Xenografted Tumor Model

Twenty-four male BALB/C nude mice (4 weeks of age, 12–14 g) were purchased from Slac Laboratory Animal Co., Ltd., Chinese Academy of Sciences (Shanghai, China) and were raised under specific pathogen-free conditions. All animal work was performed in accordance with protocols approved by the Shanghai Medical Experimental Animal Care Commission. Ethical approval was obtained from the Research Ethics Committee of Zhongshan Hospital (date of approval, 5 January 2015; permission code, 20150105082). All surgery was performed under anesthesia with sodium pentobarbital. To assess the tumorigenicity of stable CAPS1 overexpression clones, 10^7^ Huh7 or HCCLM3 cells stably transfected with control vector or CAPS1 in 0.1 mL of phosphate-buffered saline (PBS) were injected subcutaneously into the right flank of each mouse. For each cell line, six mice were recruited for the control and CAPS1 overexpression group, respectively. Tumor sizes were recorded once a week. Mice were sacrificed at 4 weeks post-injection. Tumor volume was calculated by the formula: 0.5 × *L* × *W*^2^ (*L* = length of tumor; *W* = width of tumor).

### 4.9. Statistical Analysis

Experimental data were presented as mean ± standard deviation. SPSS software (20.0; IBM, Chicago, IL, USA) was used for statistical analysis. Analysis of the association between CAPS1 expression and clinicopathological characteristics was carried out using the chi-square test or Fisher’s exact test. Student’s *t* test was used for comparison between groups. Kaplan–Meier analysis (log-rank test) was utilized for OS and TTR curves. Univariate and multivariate Cox proportional hazards regression analyses were performed to analyze the independent prognostic factors. *p* < 0.05 (two-side) was considered to be statistically significant.

## Figures and Tables

**Table 1:** Correlation between CAPS1 and clinicopathologic characteristics.

Characteristics	CAPS1 Expression in Tumor Tissue		
	Negative	Positive	*p* Value
Patients	121	20	
Gender			
Female	19	2	0.746
Male	102	18	
Age (years)			
≤52	67	11	0.975
>52	54	9	
HBsAg			
Negative	16	0	0.178
Positive	105	20	
HCV			
Negative	119	20	1.000
Positive	2	0	
ALT (units/L)			
≤75	99	20	0.081
>75	22	0	
Preoperative AFP, ng/mL			
≤20	55	8	0.649
>20	66	12	
Liver cirrhosis			
No	22	2	0.561
Yes	99	18	
BCLC stage			
A	33	5	0.832
B/C	88	15	
Tumor size (cm)			
≤5	60	9	0.704
>5	61	11	
Tumor number			
Single	90	19	0.080
Multiple	31	1	
Tumor encapsulation			
No	77	7	**0.016**
Complete	44	13	
Vascular invasion			
No	82	15	0.518
Yes	39	5	
TNM stage			
I	67	18	**0.003**
II–III	54	2	
Tumor differentiation			
I–II	114	19	1.000
III–IV	7	1	

χ^2^ tests for all the other analyses; Bold *p* values lower than 0.05 indicate statistical significance; Abbreviations: AFP: α-fetoprotein; ALT, alanine aminotransferase; BCLC: Barcelona clinic liver cancer; CAPS1: calcium-dependent activator protein for secretion 1; HBsAg, hepatitis B surface antigen; HCV, hepatitis C virus; TNM, tumor–node–metastasis.

**Table 2:** Univariate analysis of factors associated with survival and recurrence.

Variables	OS		TTR	
	Hazard Ratio (95% CI)	*p*	Hazard Ratio (95% CI)	*p*
Gender (female vs. male)	0.930	0.841	0.842	0.571
	(0.456–1.894)		(0.464–1.528)	
Age, years (≤52 vs. >52)	0.939	0.813	0.909	0.677
	(0.558–1.581)		(0.581–1.423)	
HBsAg (positive vs. negative)	0.957	0.918	0.716	0.349
	(0.410–2.232)		(0.356–1.414)	
HCV (positive vs. negative)	1.276	0.810	2.300	0.247
	(0.176–9.262)		(0.562–9.416)	
BCLC stage (A vs. B/C)	3.653	**0.003**	1.618	0.080
	(1.568–8.512)		(0.944–2.775)	
Liver cirrhosis (no vs. yes)	1.609	0.272	1.463	0.262
	(0.689–3.757)		(0.752–2.846)	
ALT, units/L (≤75 vs. >75)	1.192	0.629	1.542	0.147
	(0.584–2.430)		(0.858–2.768)	
AFP, ng/mL (≤20 vs. >20)	1.755	**0.043**	1.129	0.592
	(1.019–3.022)		(0.724–1.761)	
Tumor differentiation (I–II vs. III–IV)	1.007	0.989	2.005	0.081
	(0.364–2.786)		(0.919–4.374)	
Tumor encapsulation (complete vs. none)	0.693	0.185	0.570	**0.020**
	(0.403–1.192)		(0.356–0.914)	
Tumor size, cm (≤5 vs. >5)	2.755	**0.001**	2.152	**0.001**
	(1.527–4.968)		(1.351–3.428)	
Tumor number (single vs. multiple)	1.282	0.398	1.450	0.148
	(0.721–2.282)		(0.877–2.399)	
TNM stage (I vs. II–III)	2.776	**0.000**	2.147	**0.001**
	(1.632–4.722)		(1.377–3.348)	
Vascular invasion (no vs. yes)	1.837	**0.022**	1.887	**0.006**
	(1.019–3.092)		(1.196–2.979)	
CAPS1 in tumor tissue (negative vs. positive)	0.276	**0.014**	0.373	**0.019**
	(0.099–0.767)		(0.198–0.864)	

Cox proportional hazards regression model was used in univariate analysis; Bold *p* values less than 0.05 indicate statistical significance; Abbreviations: CI, confidence interval; HR, hazard ratio; OS, overall survival; TTR, time to recurrence.

**Table 3:** Multivariate analyses of factors associated with OS and TTR.

Variables	Hazard Ratio (95% CI)	*p* Values
^†^ OS		
BCLC stage (A vs. B/C)	1.998 (0.710–5.627)	0.190
AFP, ng/mL (≤20 vs. >20)	1.791 (1.005–3.189)	**0.048**
Tumor size, cm (≤5 vs. >5)	1.877 (0.915–3.852)	0.086
TNM stage (I vs. II–III)	1.468 (0.805–2.678)	0.210
Vascular invasion (no vs. yes)	1.195 (0.692–2.063)	0.523
CAPS1 expression in tumor tissue (negative vs. positive)	0.209 (0.064–0.682)	**0.010**
^‡^ TTR		
Tumor encapsulation (complete vs. none)	0.586 (0.355–0.968)	**0.037**
Tumor size, cm (≤5 vs. >5)	1.942 (1.145–3.294)	**0.014**
TNM stage (I vs. II–III)	1.319 (0.795–2.191)	0.284
Vascular invasion (no vs. yes)	1.401 (0.863–2.740)	0.173
CAPS1 expression in tumor tissue (negative vs. positive)	0.324 (0.143–0.733)	**0.007**

Multivariate analysis and Cox proportional hazards regression model were used. Variables were adopted for their prognostic significance by univariate analysis (*p* < 0.05). ^†^ BCLC stage, AFP, tumor size, TNM stage, vascular invasion and CAPS1 expression in tumor tissue were included in multivariate analysis for OS; ^‡^ Tumor encapsulation, tumor size, TNM stage, vascular invasion and CAPS1 expression in tumor tissue were included in multivariate analysis for TTR; Bold *p* values lower than 0.05 indicate statistical significance.

## Supplementary Materials

Supplementary materials can be found at www.mdpi.com/1422-0067/17/10/1626/s1.
