# Supplementary material for: CAPS1 Negatively Regulates Hepatocellular Carcinoma Development through Alteration of Exocytosis-Associated Tumor Microenvironment
Source: Int J Mol Sci. 2016 Sep 27;17(10):1626. doi: 10.3390/ijms17101626 (PMC5085659; doi:10.3390/ijms17101626)
Supplement: Supplementary file 1 [file ijms-17-01626-s001.pdf]

Supplementary Materials: CAPS1 Negatively Regulates Hepatocellular Carcinoma Development through Alteration of Exocytosis-Associated Tumor Microenvironment

Ruyi Xue, Wenqing Tang, Pingping Dong, Shuqiang Weng, Lijie Ma, She Chen, Taotao Liu, Xizhong Shen, Xiaowu Huang, Si Zhang and Ling Dong

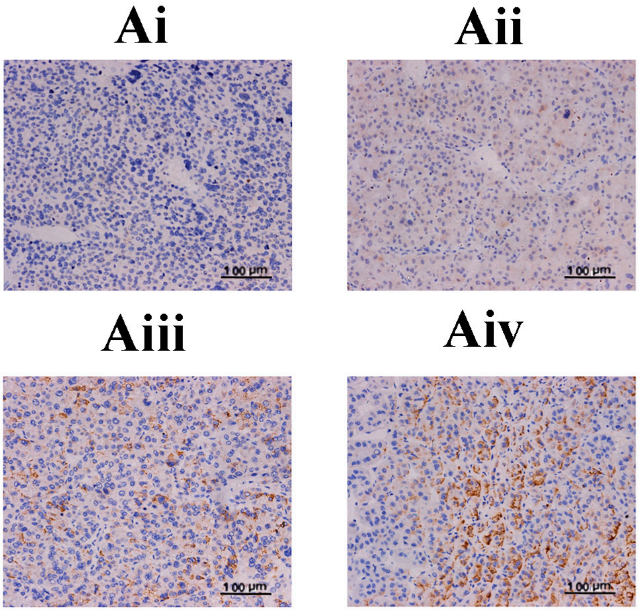

Figure S1. Representative immunostaining images of calcium-dependent activator protein for secretion 1 (CAPS1) in hepatocellular carcinoma (HCC) tissues. (**Ai**) Tumor tissues, negative staining; (**Aii**) tumor tissues, mild staining; (**Aiii**) tumor tissues, moderate staining; (**Aiv**) tumor tissues, intense staining.

Table S1. General conditions of 141 patients enrolled.

Patients	141
Gender	
Female	21
Male	120
Age, years	
≤52	78
>52	63
Hepatitis B surface antigen	
Negative	16
Positive	125
Hepatitis C virus	
Negative	139
Positive	2
ALT (units/L)	
≤75	119
>75	22
Preoperative AFP, ng/mL	
≤20	63
>20	78
Liver cirrhosis	
No	24
Yes	117
BCLC stage	
A	38
B/C	103
Tumor size (cm)	
≤5	69
>5	72
Tumor number	
Single	109
Multiple	32
Tumor encapsulation	
No	84
Complete	57
Vascular invasion	
No	97
Yes	44
TNM stage	
I	85
II–III	56
Tumor differentiation	
I–II	133
III–IV	8

ALT: alanine transaminase; AFP: α-fetoprotein; BCLC: Barcelona clinic liver cancer; TNM: tumor node metastasis.
